# Case Report: Iptacopan in a paroxysmal nocturnal hemoglobinuria patient with severe renal insufficiency

**DOI:** 10.3389/fmed.2025.1583394

**Published:** 2025-08-05

**Authors:** Ting Wu, Xiaoqin Wang

**Affiliations:** Department of Hematology, Huashan Hospital, Fudan University, Shanghai, China

**Keywords:** paroxysmal nocturnal hemoglobinuria, iptacopan, complement inhibition, extravascular hemolysis, renal insufficiency

## Abstract

Paroxysmal nocturnal hemoglobinuria (PNH) is a rare disorder characterized by complement-mediated hemolysis, thrombosis, and bone marrow failure. Iptacopan, an oral factor B inhibitor, has demonstrated efficacy in managing PNH but has not been studied in patients with severe renal insufficiency. We report a case of a PNH patient with end-stage renal disease who required renal replacement therapy and had a peritoneal dialysis catheter placed during treatment. After switching from eculizumab to iptacopan, the patient achieved transfusion independence, sustained hematologic improvement, and resolution of both intravascular and extravascular hemolysis. Iptacopan was well tolerated, with only mild adverse effects and no breakthrough hemolysis or infections. This case highlights the potential of iptacopan as a therapeutic option in PNH patients with severe renal impairment requiring dialysis.

## 1 Introduction

Paroxysmal nocturnal haemoglobinuria (PNH) is an acquired haematopoietic stem cell disease characterized by complement-mediated intravascular hemolysis, thrombosis, and bone marrow failure (1). Complement inhibitors are now the first-line treatments option for PNH, improving hemoglobin levels, reducing thrombosis risk, and prolonging survival. However, persistent anemia has become a new challenge for patients who have received C5 complement inhibitors. Iptacopan, a novel oral factor B inhibitor, was recently approved for treating PNH. However, severe renal disease patients were excluded from all of the clinical trials, and its efficacy and safety in this population are unknown. We report a case of PNH patient with severe renal insufficiency who was treated with iptacopan successfully. The patient had persisted anemia and extravascular hemolysis during eculizumab treatment. After switching to iptacopan, both intravascular and extravascular hemolysis were controlled and the patient was weaned from transfusions with mild adverse effects. No breakthrough hemolysis and infection occurred.

## 2 Case presentation

A 60-years-old man diagnosed with classic PNH was admitted to our department in January 2024. Previous medical history included diagnosis of severe aplastic anaemia at the age of forty-two treated with androgens, prednisone and cyclosporin. Serial laboratory monitoring showed that his serum creatinine level remained stable at approximately 90 μmol/L (estimated glomerular filtration rate (eGFR) 94.3 mL/min/1.73 m^2^). He had been diagnosed with PNH and received treatment with prednisone 10 years earlier. At the time of PNH diagnosis, laboratory tests revealed an elevated serum creatinine level of 143 μmol/L (eGFR 51.5 mL/min/1.73 m^2^). He presented with fatigue throughout the day and intermittently dark-colored urine. His hemoglobin fluctuated between 50 and 72 g/L. The leukocyte and platelet count remained normal. The patient’s renal function had deteriorated by December 2023, with creatinine reaching 180 μmol/L (eGFR 36.9 mL/min/1.73 m^2^) on routine evaluation. He had no history of thrombosis.

On Jan 7, 2024, he was admitted to an outside emergency department with bilateral lower extremity edema and dyspnea, which developed approximately 1 week after prednisone discontinuation during travel. Complete blood count revealed a leukocyte count of 12.54 × 10^9^/L, a hemoglobin level of 39 g/L, and a platelet count of 206 × 10^9^/L. Laboratory investigations revealed a quintessential hemolytic profile: markedly elevated lactate dehydrogenase (LDH 2500 U/L), profound reticulocytosis (14.5%), and biochemical evidence of hemoglobin catabolism featuring indirect hyperbilirubinemia (total bilirubin 78 μmol/L with indirect fraction predominating at 56 μmol/L) accompanied by undetectable haptoglobin levels (<0.1 g/L). No schistocytes were identified on peripheral blood smear. He presented with an elevated creatinine level of 992 μmol/L. Urinalysis revealed proteinuria (2+), hemoglobinuria (3+), and granular casts, without hematuria or dysmorphic red blood cells. Renal ultrasound demonstrated bilateral small kidneys with increased cortical echogenicity, findings consistent with chronic kidney disease (CKD). No hydronephrosis, calculi, or other features of postrenal obstruction were identified. Given the ultrasound evidence suggesting the irreversibility of CKD, a renal biopsy was deferred. Renal function changes are detailed in Table 1. Blood transfusions and anti-infective treatments were administered immediately along with prednisone. Renal replacement therapy with haemodialysis was also started. He became clinically stable 4 days later and then requested to see us. Laboratory investigations showed elevated creatinine 638 μmol/L, reticulocyte (Ret) 6.5%, and lactate dehydrogenase (LDH) 1,636 U/L in our hospital. His haemoglobin concentration was low at 52 g/L. After receiving *N. meningitidis* vaccine (ACYW135), he was treated with eculizumab on Jan 31, 2024. After the eculizumab induction phase (600 mg IV weekly), maintenance was continued at 900 mg IV every 2 weeks. The patient showed significant improvement with resolution of his urine color after 1 week of treatment. Laboratory data was remarkable for LDH<1.5 upper limit of normal (ULN) after 3 weeks. Iron studies during eculizumab therapy showed ferritin 155.6 μg/L and transferrin saturation 52.2%, suggesting adequate iron stores. Roxadustat was administered. However he still suffered from significant anemia and received red cell transfusions twice because of hemoglobin below 40 g/L during the whole eculizumab treatment period. Coombs’ test was positive at week 12, indicating a shift to extravascular hemolysis. Despite prescribed prednisone, the patient’s hemoglobin still dropped to 38 g/L at week 20 without any trigger events. A summary of his blood investigations is seen in Table 2.

**Table 1:** Renal function changes across different disease stages.

Date	Clinical Status	Treatment	Cr (μmol/L)	eGFR (ml/min/1.73 m^2^)
Mar 2006	Diagnosed with AA	Androgens, prednisone and cyclosporin	90	94.3
Mar 2012	Progressed to PNH	Cyclosporine discontinued, prednisone monotherapy	143	52.1
Dec 2023	Routine follow-up	Continued prednisone	180	36.9
7 Jan 2024	Acute hemolytic crisis	1 week prednisone discontinuation	992	4.8

**Table 2:** Summary of laboratory values during eculizumab introduction.

Time point	WBC (×10^9^/L)	HGB (g/L)	PLT (×10^9^/L)	Ret (%)	LDH (U/L, ULN = 250)	Cr (μmol/L)	eGFR (ml/min/1.73 m^2^)
Baseline	5.58	52	137	6.5	1,636	638	8.1
Week 1	4.15	51	127	7.4	567	577	9.1
Week 2	3.46	41	129	8.2	482	791	6.3
Week 3	4.27	44	118	7.5	373	730	6.9
Week 4	3.89	43	120	6.9	293	702	7.2
Week 6	4.71	42	127	7.7	307	755	6.6
Week 8	4.45	32	119	10.2	317	789	6.3
Week 10	3.83	41	109	7.8	394	721	7
Week 12	2.9	57	118	6.8	474	777	6.4
Week 13	3.86	60	103	6.4	457	765	6.5
Week 14	2.69	72	110	5.8	347	786	6.3
Week 16	4.73	70	70	6.6	310	817	6
Week 18	4.25	59	64	7.6	275	813	6
Week 20	3.31	38	60	13	283	813	6

WBC, white blood cell; HGB, hemoglobin; PLT, platelet; Ret, reticulocyte; LDH; lactate dehydrogenase; Cr, creatinine; eGFR; estimated glomerular filtration rate.

Following a course of streptococcus pneumoniae vaccination, he was switched to iptacopan as monotherapy, with a regimen of 200 mg administered twice daily. The Laboratory tests after 6 weeks revealed a hemoglobin level of 120 g/L, a platelet count of 87 × 10^9^/L, and a normal LDH level (Table 3). It resulted in marked improvements in fatigue. At week 11, the direct antiglobulin test turned negative, and the indirect bilirubin decreased from an initial level of 28 μmol/L to the normal range. Because of the end-stage renal disease, he had a peritoneal dialysis catheter embedded preparing for renal replacement therapy at week 16. The patient’s hemoglobin levels slightly dropped during the perioperative period and then gradually recovered. At the 29-week follow-up, we learned that he had initiated routine peritoneal dialysis treatment following an evaluation by a nephrologist. At week 37, the PNH clone on erythrocytes had expanded to 95.6%, representing a 4.5-fold increase from the pre- iptacopan baseline of 21.27%. He had been transfusion independent for 49 weeks. Throughout treatment, the patient experienced only mild headaches at the beginning of treatment without other adverse effects, breakthrough hemolysis, and infections. This case demonstrates that iptacopan can be safely used in patients with severe renal insufficiency.

**Table 3:** Laboratory values during switch from eculizumab to iptacopan.

Time point	WBC (×10^9^/L)	HGB (g/L)	PLT (×10^9^/L)	Ret (%)	LDH (U/L, ULN = 250)	Cr (μmol/L)	eGFR (ml/min/1.73 m^2^)
Last eculizumab	3.31	38	60	13	283	813	6.0
Week 2	3.09	84	66	7.22	290	637	8.1
Week 6	4.3	120	87	3.7	250	646	7.9
Week 9	5.08	116	85	1.6	212	720	7.0
Week 11	5.28	97	99	1.4	220	773	6.4
Week 15	4.31	102	115	1.0	215	856	5.7
Week 16	3.94	92	102	3.94	223	872	5.5
Week 19	4.73	98	94	2.67	213	856	5.7
Week 24	3.7	105	110	2.5	229	689	7.3
Week 29	4.25	114	121	2.8	214	789	6.2
Week 37	5.62	112	99	2.1	244	791	6.2
Week 41	4.37	108	98	2.96	230	832	5.8
Week 46	4.51	119	106	2.23	267	878	5.4
Week 49	4.91	128	122	2.28	218	774	6.3

WBC, white blood cell; HGB, hemoglobin; PLT, platelet; Ret, reticulocyte, LDH, lactate dehydrogenase; Cr, creatinine; eGFR, estimated glomerular filtration rate.

## 3 Discussion

Paroxysmal nocturnal hemoglobinuria (PNH) is an acquired clonal hematopoietic stem cell disorder characterized clinically by intravascular hemolysis, thrombosis, renal dysfunction, and fatigue (1). It has been reported that 10–30% of aplastic anemia (AA) patients after immunosuppressive therapy can progress to clinical PNH during long-term follow-up (2). The presence of PNH clones at initial diagnosis is closely associated with the progression of AA to PNH and suggests a favorable prognosis. This patient, who initially received immunotherapy following AA diagnosis, progressed to PNH 6 years later. Unfortunately, due to the lengthy disease course, PNH clone testing at initial diagnosis of AA is unavailable.

Chronic kidney disease (CKD) or acute kidney injury (AKI) is common among PNH patients. The researchers found that over half of PNH patients may develop kidney disease, with renal insufficiency serving as an independent factor for poor prognosis (3). Patients with AKI on CKD have increased thromboembolic recurrence rates and significantly higher mortality risks. Therefore, renal function should be systematically monitored. Renal insufficiency in PNH patients is caused by several factors, including chronic exposure to free haemoglobin and consuming nitric oxide, which increases haemosiderin accumulation, tubulointerstitial inflammation and kidney damage (4). This patient developed CKD stage 3 (eGFR 52.1 mL/min/1.73 m^2^) in March 2012 after transforming to classic PNH due to recurrent hemolysis. In January 2024, the patient experienced a hemolytic crisis and AKI after abandoning corticosteroids. Regrettably, renal biopsy was not performed due to the high procedural risk associated with renal atrophy. While the exact etiology of AKI could not be pathologically confirmed, the clinical course and historical data suggested acute-on-chronic renal failure secondary to hemolytic crisis in a patient with pre-existing CKD. Earlier intervention with plasma exchange or high-volume hemoperfusion might have preserved residual renal function.

Eculizumab can significantly improve CKD stages within 6 months, with higher improvement rates in CKD stages 1–2 compared to stages 3–5 (5). The patient was already in CKD stage 5 at the initial of eculizumab therapy, his renal function was not restored after 21 weeks treatment, highlighting the importance of early complement inhibitor therapy, especially for PNH patients with renal impairment. Persistent anemia remains a challenge for PNH patients who are treated with eculizumab, with only 20–30% of patients attaining normal hemoglobin levels (6). Over 50% of patients still exhibited mild to moderate PNH-related symptoms, and 20% of patients required transfusions (7). The patient had an increase in the hemoglobin level that transiently reached a maximum of 72 g/L during eculizumab therapy. He experienced severe anemia and required red cell transfusions. Extravascular hemolysis occurred at week 13, leading to a switch to iptacopan monotherapy.

Iptacopan is an oral proximal complement inhibitor targeting factor B in the alternative pathway. It was approved for adult PNH patients who have not previously received complement inhibitors in China. Phase II trials have shown that iptacopan eliminated intravascular hemolysis in PNH patients and reduced extravascular hemolysis in patients with persistent anemia who had been treated with C5 complement inhibitors (8, 9). Two pivotal Phase III trials further demonstrated that iptacopan improved hemoglobin levels in patients with C5 complement inhibitors (APPLY-PNH) and patients who had not undergone complement inhibitors (APPOINT-PNH), reduced fatigue, lowered reticulocyte and bilirubin levels, and brought LDH <1.5 ULN (10). Although PNH clone increased in patients with iptacopan, only two of the 62 patients experienced breakthrough hemolysis in APPLY-PNH trial, and no clinical breakthrough hemolysis occurred in APPOINT-PNH trial. Common side effects of iptacopan include headache, infections, and diarrhea (11). FDA has approved iptacopan for a new indication to reduce proteinuria in adults with IgA nephropathy on August 8, 2024. However, severe renal disease patients were excluded from all of the clinical trials, and its efficacy and safety in this population are unknown. Drug metabolism studies indicate that hepatic metabolism is the primary clearance pathway for iptacopan, suggesting minimal impact of renal impairment on drug metabolism (12). After switching to iptacopan, this patient had a significant increase in hemoglobin and platelet levels without breakthrough hemolysis or thromboembolic events. The adverse events were limited to mild headaches during initial treatment.

This is the first case of a PNH patient with severe renal insufficiency who was successfully treated with iptacopan. It will provide a basis for expanding indications of iptacopan in the subsequent clinical trials. However, a large-scale study with longer follow-ups is required to further confirm the clinical efficacy of iptacopan in PNH patients with severe renal insufficiency and its potential of renal function improvement. Additionally, iptacopan significantly increased the PNH clone proportion, and strong complement-amplifying events such as infections, trauma, and major surgery may heighten the risk of severe breakthrough hemolysis and thrombosis. Further studies are needed to manage pharmacodynamic breakthrough hemolysis, thrombosis risk, and to assess long-term safety concerning infection, fertility, and other factors.

## Data Availability

The original contributions presented in the study are included in the article/supplementary material, further inquiries can be directed to the corresponding author.
